# Fine-Tuning the Activation Mode of an 1,3-Indandione-Based Ruthenium(II)-Cymene Half-Sandwich Complex by Variation of Its Leaving Group

**DOI:** 10.3390/molecules24132373

**Published:** 2019-06-27

**Authors:** Stephan Mokesch, Daniela Schwarz, Michaela Hejl, Matthias H. M. Klose, Alexander Roller, Michael A. Jakupec, Wolfgang Kandioller, Bernhard K. Keppler

**Affiliations:** 1 Institute of Inorganic Chemistry, Faculty of Chemistry, University of Vienna, Waehringer Straße 42, A-1090 Vienna, Austria; 2 Research Cluster “Translational Cancer Therapy Research”, University of Vienna, Waehringer Straße 42, A-1090 Vienna, Austria

**Keywords:** half-sandwich complexes, ruthenium(II)–arene complexes, leaving group variation, anticancer drugs

## Abstract

Fine-tuning of the properties of a recently reported 1,3-indandione-based organoruthenium complex is attempted to optimize the stability under physiological conditions. Previous work has shown its capacity of inhibiting topoisomerase IIα; however, fast aquation leads to undesired reactions and ligand cleavage in the blood stream before the tumor tissue is reached. Exchange of the chlorido ligand for six different *N*-donor ligands resulted in new analogs that were stable at pH 7.4 and 8.5. Only a lowered pH level, as encountered in the extracellular space of the tumor tissue, was capable of aquating the complexes. The 50% inhibitory concentration (IC_50_) values in three human cancer cell lines differed only slightly, and their dependence on the utilized leaving group was smaller than what would be expected from their differences in cellular accumulation, but in accordance with the very minor variation revealed in measurements of the complexes’ lipophilicity.

## 1. Introduction

**Figure 1:**
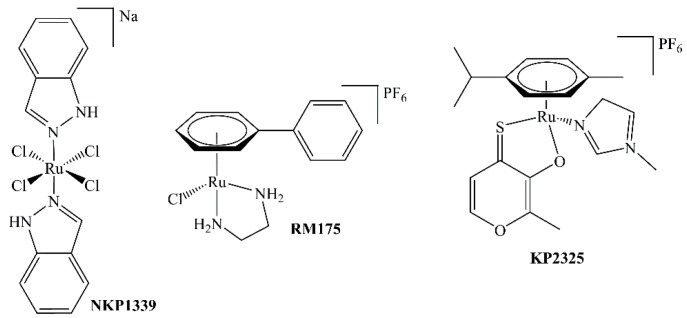
Structures of NKP1339, RM175, and KP2325.

In 1976, the ruthenium complex fac-[Ru(NH_3_)_3_Cl_3_] was reported to cause filamentous growth of *E. coli*, indicative of an inhibitory effect on cell division; however, poor aqueous solubility hindered further advances [1]. A major step toward clinical use of ruthenium-based metallodrugs is the development of NAMI-A and IT-139 (NKP-1339, Figure 1), two octahedral Ru^III^ complexes which are thought to be activated by reduction to the more reactive Ru^II^ species in the hypoxic tumor milieu [2]. IT-139 is on the verge of phase II clinical trials, whereas the clinical development of NAMI-A has been terminated [3].

Besides Ru^III^ complexes, the class of organometallic Ru^II^ compounds has shown promising results with novel modes of action compared to clinically established platinum drugs, depending on the attached ligand systems. These so-called piano-stool complexes consist of a facially coordinated arene (e.g., cym = η^6^-p-cymene) and the remaining three coordination sites are in most cases occupied by one bidentate and one labile monodentate ligand. Under proper conditions, the monodentate leaving group is substituted by a water molecule, yielding the biologically active species. The best-known complexes of this compound class are RAPTA-C and RM175 [4,5]. RM175 is a half-sandwich complex where biphenyl (bip) is the facially bound arene, ethylenediamine is the *N,N*-chelating ligand, and the last valence is occupied by a chlorido ligand (see Figure 1). This positively charged complex can interact with DNA both in a hydrophobic manner (by the bip moiety) and by covalent binding to guanine residues. However, there is no clear indication as of yet whether RM175 exhibits selectivity for tumor cells over normal cells [6]. A new trend is the exploitation of possible synergistic effects and increased cytotoxic activity by synthesizing complexes of chelating agents that have biological activity on their own, e.g., derivatives of naphthoquinones [7], flavonoids [8], indoloquinolines [9], or thiomaltol (see Figure 1) [10,11]. Naphthoquinones, for example, are able to generate reactive oxygen species via redox-cycling [12]. 1,3-Indandion-2-carboxamides, on the other hand, have interesting topoisomerase-inhibiting properties [13,14].

However, the major drawback for many organoruthenium complexes is the insufficient stability under physiological conditions, preventing these active complexes from reaching the tumor tissue. Experiments in our working group showed rapid aquation, especially for complexes bearing an *O*,*O*-coordination motif, which would lead to premature reaction with biomolecules in the blood stream.

Substitution of the chlorido leaving group by different *N*-donors might be a possible solution and provides many advantages. Heterocyclic *N*-donor ligands bind stronger to this type of organometallics and are, therefore, less easily replaced by water. Along with this increased stability, the aqueous solubility, the lipophilicity, and the cell permeability can be increased [11,15,16]. Additionally, this approach might enable pH-dependent activation in the hypoxic tumor tissue. The selectivity of antitumor organometallics with chlorido leaving groups hinges on the difference in chloride concentration [17]. However, within the tumor tissue, a lower extracellular pH level is usually encountered [18]. By carefully considering this fact, we chose *N*-donors which can be protonated and subsequently cleaved off at this lowered pH level, leading to the formation of the more reactive aqua complexes, ready to interact with biological binding partners, i.e., proteins or DNA [11]. Half-sandwich ruthenium azole complexes have been synthesized by numerous working groups in the past. Vock et al. proposed a combination of the activity of Sadler’s [Ru(cym)(en)Cl] complex (en = ethylenediamine) with that of NAMI-A [19]. Apart from the ruthenium field, *N*-donors have been used in the synthesis of iron-based [20], osmium-based [21], and platinum-based antitumor compounds [22]. Choosing pyridine as a *N*-donor adds the possibility of light activation, as utilized in photoactivated chemotherapy (PACT) [23,24]. Another approach is the exchange of the labile leaving group by trichlorogermyl, which also resulted in an increased stability and cytotoxicity [25].

In our previous work, we have shown that organometallic 1,3-indanones exhibit promising antiproliferative properties and the capacity of inhibiting topoisomerase IIα, a key enzyme in all major DNA functions [26]. In this study, we modified the most promising candidate and substituted the chlorido leaving group with different *N*-donors. The aim was to increase the stability as well as the solubility. Additionally, a structure–activity relationship study with regard to the impact of the different leaving groups on cytotoxicity and cellular accumulation was attempted.

## 2. Results and Discussion

### 2.1. Synthesis and Characterization

**Scheme 1:**
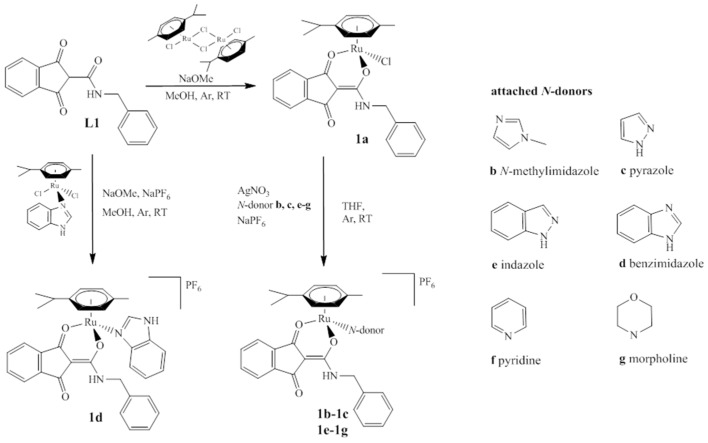
Synthesis of chlorido complexes 1a and its derivatives 1b–g bearing different monodentate *N*-donors (see right). The organometallics were obtained either by reaction of L1 with benzimidazole precursor (1d) or by exchanging the chlorido group of 1a yielding compounds **1b**, **1c**, **1e**–**g**.

A series of organometallics was synthesized, in accordance with Scheme 1, starting from the previously reported complex [(chlorido)(1,3-dioxo-κO1-1H-inden-2(3H)-ylidene)(benzylamino)methanolato-κO2)](η^6^-p-cymene) ruthenium(II) (complex 1a) [26]. After dissolving in THF, the chlorido ligand was abstracted by an equimolar amount of AgNO_3_. The appropriate *N*-donor molecules (i.e., *N*-methylimidazole (MeImid) (complex 1b), pyrazole (HPz) (complex 1c), indazole (HInd) (complex 1e), pyridine (Pyr) (complex 1f), and morpholine (Morph) (complex 1g) were added, followed by addition of NaPF_6_. Racemic mixtures of the organometallics were obtained in good yields by removal of sodium chloride and precipitation from DCM/Et_2_O. In contrast, the benzimidazole (BIm) complex (complex 1d) was synthesized via a different route. The ligand L1 2-((benzylamino)(hydroxy)methylene)-1H-indene-1,3(2H)-dione [26] was deprotonated by NaOMe and precursor [(dichlorido)(κN1-benzimidazole)(η^6^-p-cymene)] ruthenium(II) [19] was added. The complex was again precipitated in moderate yield upon treatment with NaPF_6_. Formation and purity of the complexes was confirmed by elemental analysis, 1D/2D-NMR measurements, and ESI-MS. Furthermore, the solubility in a minimal essential medium (MEM; 1% DMSO) and the decomposition point of complexes 1b–1g were determined.

^1^H and ^13^C-NMR measurements clearly confirmed the successful replacement of the chlorido ligand by the respective *N*-donor, due to small shifts of the coordinated heterocycle and the facially coordinated *p*-cymene ring. As observed for the chlorido complex (complex 1a), the methylene protons of the benzyl group were diastereotopic for complexes 1b–g leading to two separate signals with geminal couplings of about 15 Hz. In addition, four diastereotopic signals of the *p*-cymene ring protons and two partially overlapping doublets of the isopropyl group were recorded, which can be explained by the presence of a chiral metal center. The same behavior was found in ^13^C experiments where six distinct signals for the carbon atoms of the *p*-cymene ring and the two peaks for the isopropyl methyl groups were recorded. 

**Figure 2:**
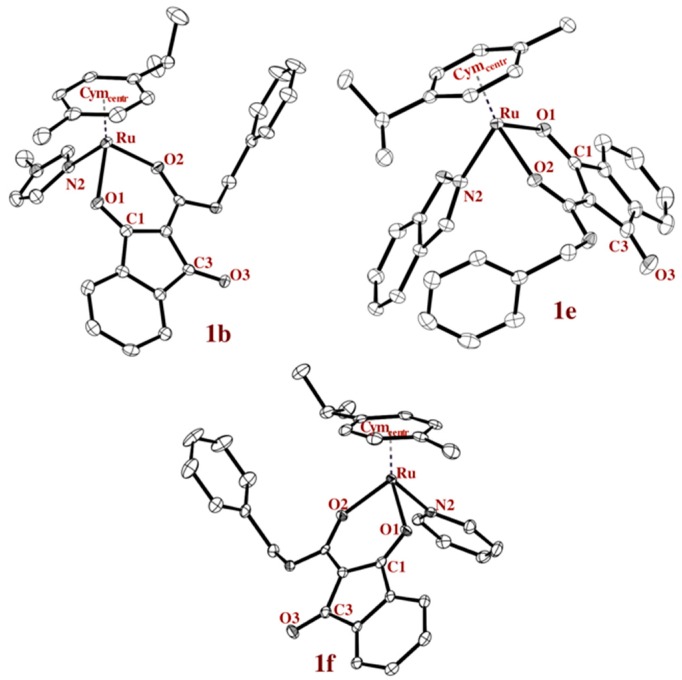
ORTEP representations of complexes 1b (MeIm), 1e (HInd), and 1f (Pyr) drawn at the 50% probability level (hydrogen atoms and counter ions are omitted for clarity).

Single crystals of complexes 1b–1f suitable for X-ray diffraction analysis were obtained by slow diffusion method and the solid state structures of three examples are presented in Figure 2. It has to be mentioned that suitable crystals of complexes 1c, 1e, and 1f could only be obtained by exchanging the hexafluorophosphate with triflate. Crystallographic parameters, CCDC numbers and the other two crystal structures are given in the supporting information (Chart S1 and Tables S1–S11, Supplementary Materials). The most important bond lengths and angles are listed in Table 1 and compared to the chlorido analogue (complex 1a). For instance, the distance of the chlorido ligand to the ruthenium center, as well as the distance to the respective *N*-atom, is in good accordance with similar organoruthenium compounds [11].

In chlorido complex (complex 1a), the bond length of the coordinated keto-function (1.267 Å) is longer than that of the other one (1.222 Å), as is expected. However, the higher electron density of the ruthenium center, due to the lower electronegativity of the *N*-donors, as opposed to the chlorido ligand, leads to more similar keto bond lengths. The distance of the metal center to the stabilizing aromatic ring is slightly shorter in complex 1a than for complexes 1b–1f.

### 2.2. Lipophilicity

It stands to reason that the higher the lipophilicity, the higher the cell accumulation; as far as passive diffusion is concerned, the higher efficiency with which normal cell function is compromised. As a consequence, lipophilicity is an important factor in drug development, described by the partition coefficient log*P*, which is routinely determined by the distribution of a compound in 1-octanol/water. However, 1-octanol, a solvent said to closely mimic the solubilizing behavior of cell membranes, forms emulsions with water [27]. Additionally, the shake-flask method, seemingly easy, requires long sample preparation, has a low sample throughput, and lacks reproducibility, as it hinges on exact temperature control and consistent equilibration time allotted for every analyte. The time-consuming nature of this method may also lead to decomposition of the analyte. A concentration-, flow rate-, and column length-independent method is the determination of the capacity factor (k) via HPLC. The capacity factor is a measure for the equilibrium concentration between a polar phase (eluent) and an apolar phase (C18 column coating) and higher resemblance to the partition coefficient in water was found (1-octanol will be achieved if the eluent is 100% water). Extrapolation to this theoretical setup results in the highly comparable factor log*k*_w_. The percentage of methanol in the eluent at which log*k* is equal to zero is Φ_0_ [27,28,29].

Both parameters (log*k*_w_ and Φ_0_) were determined for complexes 1a–g (shown in Scheme 1) (see Table 2). A minimum of R_2_ = 0.998 was obtained for every capacity factor. The most hydrophilic compound was the morpholine complex 1g (log*k*_w_ = 5.14 and Φ_0_ = 59.73) and the most lipophilic one was complex 1e (HInd, log*k*_w_ = 5.94 and Φ_0_ = 68.88). In aqueous systems, the chlorido complex 1a hydrolyzed quickly to the more reactive aqua complex. Therefore, the determined lipophilicity of complex 1a (log*k*_w_ = 5.41 and Φ_0_ = 63.19) can be attributed to the aquated species and was situated between these limits (see respective tables in Supplementary Materials, Tables S12 and S13).

In general, it can be seen that the lipophilicity-determining factors are the *O,O*-chelating ligand and the facial arene, as all compounds behaved quite similarly. Due to the small numeric differences in the measured lipophilicity factors, complexes 1a–1c and 1f exhibited a different lipophilic ranking, depending on whether log*k*_w_ or Φ_0_ was the observed variable. It is noteworthy that said lipophilicities varied within 20% and 30% of the entire measured range for all seven compounds.

### 2.3. pH-Dependent Stability

The stability under physiological conditions is a vital part of the process of developing a new drug. Additionally, *N*-donor leaving groups equip piano-stool complexes with an elegant pH-switch. Theoretically, they can be protonated at lower pH values, as found in the extracellular matrix of the tumor tissue, and are therefore cleaved off. The resulting aqua complex is highly reactive and able to interact with biological targets. To put this theory to the test, the stability of complexes 1a–g was investigated at different pH values, i.e., 5.5, 6.5, 7.4, and 8.5. Spectra were recorded over a period of 24 h, with 40 µM solutions (1% DMSO in water) in the range of 250–700 nm. The chosen pH values represented the physiologically relevant conditions; a pH value of 8.5 is the highest pH found in the human body (in the duodenum), a pH value of 7.4 is the average pH of human blood, and a pH value of 6.5 is the pH found in the extracellular matrix of solid tumors [30]. 

All compounds proved to be stable at pH 7.4 (blood pH, Figure S2, Supplementary Materials) and pH 8.5 (duodenal pH, Figure S1, Supplementary Materials). Only complexes 1d (BIm) and 1e (HInd) precipitated to some extent at pH 7.4 (loss of absorbance without change in spectrum, Figure S3, Supplementary Materials). At pH 6.5 (tumor pH, Figure S4, Supplementary Materials), all compounds remained stable for 24 h as well. At pH 5.5, all test compounds lost their *N*-donor ligand within the first hour and the active aqua complex analogous to complex 1a was released, confirming our approach. Since the leaving groups of complexes 1b–g were fully cleaved off at pH 5.5, the organometallics should be able to reach the tumor environment intact, where only a minor amount was first activated. However; the aqua complex was readily removed by reaction with biological nucleophiles and therefore the equilibrium was shifted towards the aquation of complexes 1b–g.

Since the pH value of the stock solution is lowered with acetic acid, not the aqua species was observed, but rather a labile acetato complex. As can be seen in Figure S5 (Supplementary Materials), it exhibits a drastically different absorption spectrum and therefore functions as an unambiguous indicator for drug activation. The acetato ligand is a hard ligand and is not favored by the soft Ru^II^ center ion. At pH 8.5, no acetato adduct was detected, even after addition of 10 eq NaOAc, allowing for the conclusion that only if the stable *N*-donor has been protonated and has cleaved off, the acetato complex can form.

**Figure 3:**
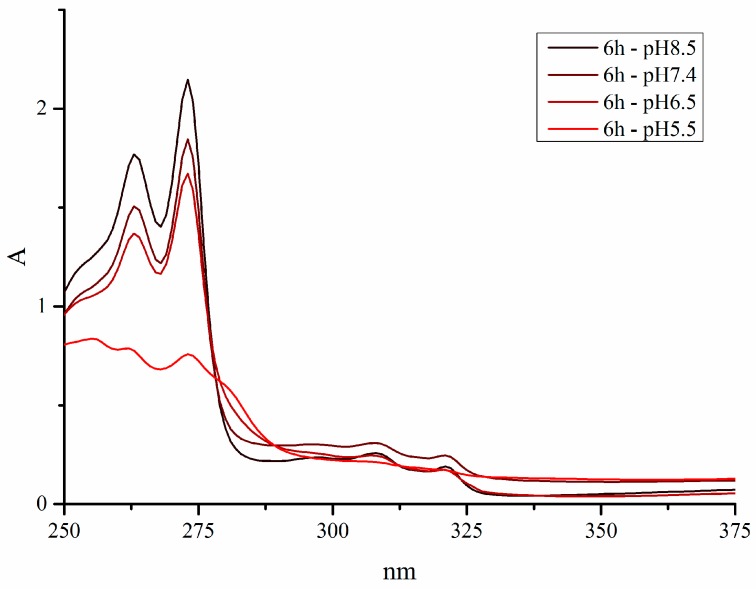
UV-VIS spectra of pyridine complex 1f at pH 5.5, 6.5, 7.4, and 8.5 in 1% (*v*/*v*) DMSO/H_2_O (0.9% NaCl).

Furthermore, spectra were recorded in solutions with and without chloride ions (see Figure 3 for the spectra of pyridine complex 1f). Since the investigated organometallics behaved in the same manner in both conditions, it is obvious that their method of action is chloride-independent as opposed to platinum compounds.

### 2.4. Light Activation of Complex 1f

**Figure 4:**
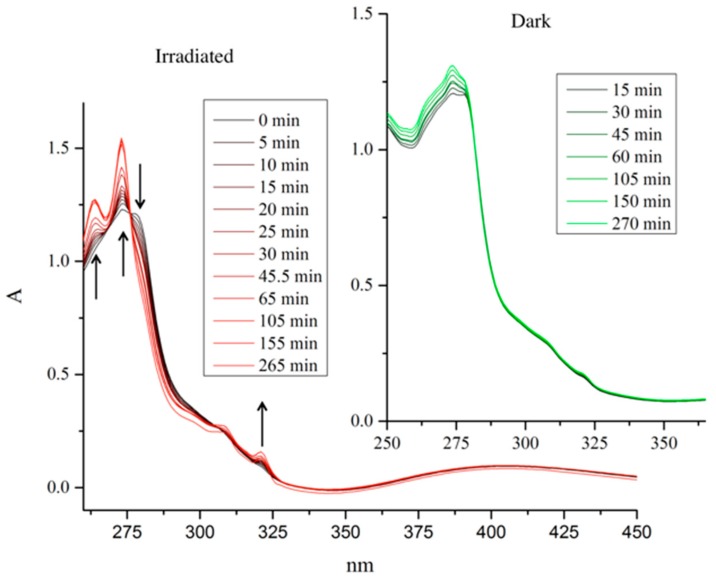
UV-VIS spectra of pyridine complex 1f in MeOH in the light (red) and in the dark (green).

Photoactivated chemotherapy (PACT) is an up-and-coming area of research. The idea is that transition metal complexes undergo ligand dissociation (once they have accumulated within the tumor tissue) by light irradiation [24]. Ligand systems based on the pyridine motif are especially suited for this purpose. Therefore, pyridine complex 1f was investigated for its potential to be activated upon light irradiation. The observed peak at 278 nm slowly decreased and peaks at 264, 273, and 321 nm increased. After 75 min, no more change was observed, signifying the completion of the reaction. Two well-separated isosbestic points were observed at 276 and 306 nm, indicating a well-defined hydrolysis reaction (see Figure 4). Apart from complex 1f, no other compound showed any leaving group cleavage upon irradiation.

### 2.5. Biological Activity and Cellular Accumulation Studies

The antiproliferative activity of the complexes was studied in four human cancer cell lines in vitro. In each cell line, differences between the cytotoxic potencies of the compounds are rather small (Table 3). In the more sensitive cell lines SW480 and CH1/PA-1, differences are slightly more pronounced, with 50% inhibitory concentration (IC_50_) values ranging from 6 to 17 μM in the former and from 8 to 16 μM in the latter. Overall, the IC_50_ values are markedly lower than those of compounds with different 1,3-dioxoindan-2-carboxamide derivatives as the chelating ligand, which have been previously investigated alongside with the chlorido complex 1a that acts as the basis for this *N*-donor variation study [26]. While complex 1a is among the complexes with the least pronounced antiproliferative effects here, increases in activity brought about by the *N*-donor ligands never exceeded a factor of 2 in terms of IC_50_ value. Due to the small differences, there is no evident correlation between lipophilicity and antiproliferative activity (Figure S14, Supplementary Materials).

Ruthenium accumulation was measured by means of inductively coupled plasma mass spectrometry (ICP-MS) upon 2 h exposure of SW480 cells to 50 µM of each compound. Given the fact that differences in cytotoxicity are not pronounced, it is rather remarkable that cellular accumulation is quite different (Table 3). The benzimidazole complex 1d yielded the highest cellular ruthenium values (>400 fg/cell), while that of the chlorido analog 1a is almost fiftyfold lower (<10 fg/cell). Generally, all *N*-donor complexes were accumulated to a higher extent (at least eight times) than the latter. Thus, the 2 h endpoint measurements suggested that azole complexes require higher cellular uptake than the chlorido analog for a comparable effect. Results for the six azole complexes differed only by factors of up to six among each other, with complexes having IC_50_ values of <10 µM in SW480 cells (complexes 1d, 1f, and 1g) showing higher accumulation than those with IC_50_ values of >10 µM (complexes 1b, 1c, and 1e).

## 3. Materials and Methods

### 3.1. Materials

All solvents were purchased from commercial sources and distilled prior to use. 1-Methylimidazole (99%, Sigma-Aldrich, Vienna, Austria), pyrazole (98%, Sigma-Aldrich, Vienna, Austria), benzimidazole (≥98%, Sigma-Aldrich, Vienna, Austria), indazole (≥98%, Polivalent95, Chisinau, Moldavia), pyridine (>99%, Fisher Scientific, Vienna, Austria), morpholine (≥99.5%, Aldrich, Vienna, Austria), silver nitrate (≥99.5%, Aldrich, Vienna, Austria), and sodium hexafluorophosphate (95.5%, Fisher Scientific, Vienna, Austria) were purchased and used without further purification. 2-((Benzylamino)(hydroxy)methylene)-1H-indene-1,3(2H)-dione, [(Chlorido)(1,3-dioxo-κO1-1H-inden-2(3H)-ylidene)(benzylamino)methanolato-κO2)](*η*^6^-p-cymene)ruthenium(II) [26] (complex 1a), and [(dichlorido)(κN1-Benzimidazole)(*η*^6^-p-cymene)] ruthenium(II) [19] were synthesized as described elsewhere.

Melting points were determined with a Büchi Melting Point M-560. The solubility was determined by dissolving the compound in DMSO and subsequent dilution to a final concentration of 1% DMSO/MEM solution (minimal essential medium) and 1% DMSO/MSP solution (medium with serum proteins). The highest concentrated dilution, where no precipitation of the compound occurred, was the determined solubility. The NMR spectra were recorded at 25 °C using a Bruker FT-NMR spectrometer Avance III^TM^ 500 MHz. ^1^H-NMR spectra were measured at 500.10 MHz and ^13^C-NMR spectra were recorded at 125.75 MHz in deuterated methanol (CD_3_OD). 2D-NMR spectra were measured using a gradient-enhanced mode. CHN elemental analyses were performed with a Eurovector EA3000 Elemental Analyzer in the microanalytical laboratory of the University of Vienna. Single crystals of complexes 1b–1f suitable for X-ray diffraction analysis were grown by precipitation from DCM/Et_2_O at 4 °C. The X-ray intensity data were measured on Bruker D8 Venture or X8 APEX II diffractometer equipped with multilayer monochromators, Mo K/α INCOATEC microfocus sealed tubes and Kryoflex cooling devices (Bruker, Karlsruhe, Germany). The structures were solved by direct methods and refined by full-matrix least-squares techniques. Non-hydrogen atoms were refined with anisotropic displacement parameters. Hydrogen atoms were inserted at calculated positions and refined with a riding model separately as rotating groups. The following software was used: Bruker SAINT software package using a narrow-frame algorithm for frame integration, SADABS [33] for absorption correction, OLEX2 [34] for structure solution, refinement, molecular diagrams and graphical user-interface, Shelxle [35] for refinement and graphical user-interface, SHELXS-2013 [36] for structure solution, SHELXL-2013 [37] for refinement, and Platon [38] for symmetry check. Electronspray ionization mass spectra were recorded on a Bruker AmaZon SL ion trap mass spectrometer (Bruker Daltonics GmbH, Hamburg, Germany).

### 3.2. General Ligand Exchange Procedure

A solution of complex 1a (1 eq) and silver nitrate (1.4 eq) were suspended in THF and stirred for 2 h at room temperature under an argon atmosphere. Then, the appropriate *N*-donor (1.2 eq) was added and the mixture was stirred for further 2 h under the same conditions followed by addition of sodium hexafluorophosphate (1.3 eq). After another 2 h under the same conditions, the THF was removed and the residue remained in DCM. After a filtration step, the solution was concentrated and precipitated from DCM/Et_2_O.

### 3.3. Synthesis of Complexes 1b–g

[(κN2-*N*-Methylimidazole)(1,3-dioxo-κO1-1H-inden-2(3H)-ylidene)(benzylamino)methanolato-κO2)](*η*^6^-p-cymene)ruthenium(II) hexafluorophosphate (complex 1b)

The synthesis was performed according to the general procedure for ligand exchange, using complex 1a (110 mg, 200 µmol), silver nitrate (48 mg, 280 µmol), *N*-methylimidazole (20 µL, 240 µmol), and sodium hexafluorophosphate (42 mg, 260 µmol), yielding yellow crystals (120 mg, 81%). m.p.: >187 °C (decomp.), solubility: 0.30 mg/mL = 0.40 mM (MEM, 1% DMSO); ^1^H-NMR (500.10 MHz, MeOD) δ: 1.29 (d, ^3^J(H,H) = 7 Hz, 3H, CH_3_-f), 1.31 (d, ^3^J(H,H) = 7 Hz, 3H, CH_3_-f), 2.13 (s, 3H, CH_3_-g), 2.71–2.76 (m, 1H, He), 3.65 (s, 3H, H10′), 4.61 (d, ^2^J(H,H) = 15 Hz, H, H2′), 4.76 (d, ^2^J(H,H) = 15 Hz, H, H2′), 5.58–5.63 (m, 2H, Hb), 5.74–5.81 (m, 2H, Hc), 6.67 (s, 1H, H7′), 7.07 (s, 1H, H8′), 7.32–7.46 (m, 5H, H4′, H5′, H6′), 7.48–7.63 (m, 5H, H4, H5, H6, H7, H9′), 9.10 (t, ^3^J(H,H) = 6 Hz, 1H, NH); ^13^C-NMR (125.75 MHz, MeOD) δ: 17.7 (CH_3_-g), 22.3 and 22.6 (2CH_3_-f), 32.0 (Ce), 34.9 (C10′), 44.4 (C2′), 82.3 and 82.5 (Cb), 84.5 and 84.7 (Cc), 98.5 (C2), 99.2 (Cd), 103.5 (Ca), 121.8 (C4, C7), 123.7 (C8′), 128.4 (C5, C6), 128.5 (C4′, C6′), 129.8 (C7′), 130.0 (C5′), 134.1 (C9′), 136.7 (C7a), 138.4 (C3a), 140.5 (C3′), 167.0 (C1′), 192.3 (C3), 193.6 (C1); *m*/*z* 596.148, m^th^: 596.149; elemental analysis calcd. for C_31_H_32_F_6_N_3_O_3_PRu·0.5H_2_O: C, 49.67; H, 4.44; N, 5.61%; found: C, 49.66; H, 4.24; N, 5.76%.

[(κN2-1H-Pyrazole)(1,3-dioxo-κO1-1H-inden-2(3H)-ylidene)(benzylamino)methanolato-κO2)](*η*^6^-p-cymene)ruthenium(II) hexafluorophosphate (complex 1c)

The synthesis was performed according to the general procedure for ligand exchange, using complex 1a (110 mg, 200 µmol), silver nitrate (48 mg, 280 µmol), pyrazole (16 mg, 240 µmol), and sodium hexafluorophosphate (42 mg, 260 µmol), yielding yellow crystals (102 mg, 70%). m.p.: >137 °C (decomp.), solubility: 0.28 mg/mL = 0.39 mM (MEM, 1% DMSO); ^1^H-NMR (500.10 MHz, MeOD) δ: 1.22 (d, ^3^J(H,H) = 7 Hz, 3H, CH_3_-f), 1.27 (d, ^3^J(H,H) = 7 Hz, 3H, CH_3_-f), 2.06 (s, 3H, CH_3_-g), 2.61–2.69 (m, 1H, He), 4.69 (d, ^2^J(H,H) = 15 Hz, H, H2′), 4.73 (d, ^2^J(H,H) = 15 Hz, H, H2′), 5.60–5.67 (m, 2H, Hb), 5.80–5.86 (m, 2H, Hc), 6.37 (s, 1H, H8′), 7.17 (s, 1H, H9′), 7.32–7.68 (m, 9H, H4, H5, H6, H7, H4′, H5′, H6′), 7.83 (s, 1H, H7′), 7.98–8.00 (m, 1H, NH-HPz), 9.09 (t, ^3^J(H,H) = 6 Hz, 1H, NH), ^13^C-NMR (125.75 MHz, MeOD) δ: 17.9 (CH_3_-g), 22.4 and 22.5 (2CH_3_-f), 32.1 (Ce), 44.4 (C2′), 81.4 and 81.6 (Cb), 85.4 (Cc), 98.4 (C2), 100.7 (Cd), 103.7 (Ca), 108.3 (C8′), 121.9 and 122.1 (C4, C7), 128.3 (C5, C6), 128.5 (C4′, C6′), 129.8 (C5′), 134.5 (C7′), 136.5 (C7a), 138.4 (C3a), 140.3 (C3′), 143.0 (C9′), 166.8 (C1′), 192.2 (C3), 193.5 (C1); *m*/*z* 582.133, m^th^: 582.132; elemental analysis calcd. for C_30_H_30_F_6_N_3_O_3_PRu: C, 49.59; H, 4.16; N, 5.78%; found: C, 49.36; H, 3.99; N, 5.76%.

[(κN2-Benzimidazole)(1,3-dioxo-κO1-1H-inden-2(3H)-ylidene)(benzylamino)methanolato-κO2)](*η*^6^-p-cymene)ruthenium(II) hexafluorophosphate (complex 1d) 

[(Dichlorido)(κN1-Benzimidazole)(*η*^6^-p-cymene)]ruthenium(II) [19] (109 mg, 260 µmol) was added to a solution of *N*-benzyl-1,3-dioxo-2,3-dihydro-1H-inden-2-carboxamide [25] (80 mg, 290 µmol) and sodium methanolate (20 mg, 315 µmol) in methanol (30 mL). The mixture was irradiated for 5 min at 50 °C. Then, sodium hexafluorophosphate (63 mg, 370 µmol) was added and the mixture was stirred for further 4.5 h at room temperature. The precipitate was filtered off and washed with n-Hexane. This afforded the product as a brown powder (129 mg, 65%). m.p.: >216 °C (decomp.), solubility: 0.29 mg/mL = 0.37 mM (MEM, 1% DMSO); ^1^H-NMR (500.10 MHz, MeOD) δ: 1.29 (d, ^3^J(H,H) = 7 Hz, 3H, CH_3_-f), 1.33 (d, ^3^J(H,H) = 7 Hz, 3H, CH_3_-f), 2.06 (s, 3H, CH_3_-g), 2.74–2.82 (m, 1H, He), 4.68 (d, ^2^J(H,H) = 15 Hz, H, H2′), 4.73 (d, ^2^J(H,H) = 15 Hz, H, H2′), 5.51 (s, 1H, NH-BenzIm), 5.69–5.77 (m, 2H, Hb), 5.87–6.00 (m, 2H, Hc), 7.33–7.68 (m, 13H, H4, H5, H6, H7, H4′, H5′, H6′, H8′, H9′, H10′, H11′), 7.92–7.95 (m, 1H, H7′), 9.00 (t, ^3^J(H,H) = 6 Hz, 1H, NH); ^13^C-NMR (125.75 MHz, MeOD) δ: 18.1 (CH_3_-g), 22.5 and 22.6 (2CH_3_-f), 32.2 (Ce), 44.5 (C2′), 81.6 and 81.7 (Cb), 84.8 and 85.2 (Cc), 98.3 (C2), 99.8 (Cd), 104.4 (Ca), 119.1 (C7′), 114.2, 121.8, 121.9, 125.0, 125.8, 128.5, 128.6, 129.9, 134.0, 143.7 (C4, C5, C6, C7, C4′, C5′, C6′, C8′, C9′, C10′, C11′), 134.2, 136.5, 138.2, 140.4, 141.4 (C3a, C7a, C3′, C8a’, C12a’), 166.6 (C1′), 192.1 (C3), 193.4 (C1); *m*/*z* 632.149, m^th^: 632.149; elemental analysis calcd. for C_34_H_32_F_6_N_3_O_3_PRu: C, 52.58; H, 4.15; N, 5.41%; found: C, 52.34; H, 3.82; N, 5.40%.

[(κN2-1H-Indazole)(1,3-dioxo-κO1-1H-inden-2(3H)-ylidene)(benzylamino)methanolato-κO2)](*η*^6^-p-cymene)ruthenium(II) hexafluorophosphate (complex 1e)

The synthesis was performed according to the general procedure for ligand exchange, using complex 1a (110 mg, 200 µmol), silver nitrate (48 mg, 280 µmol), indazole (28 mg, 240 µmol), and sodium hexafluorophosphate (42 mg, 260 µmol), yielding yellow crystals (120 mg, 81%). m.p.: >190 °C (decomp.), solubility: 0.06 mg/mL = 0.08 mM (MEM, 1% DMSO); ^1^H-NMR (500.10 MHz, MeOD) δ: 1.29 (d, ^3^J(H,H) = 7 Hz, 3H, CH_3_-f), 1.31 (d, ^3^J(H,H) = 7 Hz, 3H, CH_3_-f), 2.11 (s, 3H, CH_3_-g), 2.71–2.78 (m, 1H, He), 4.71 (dd, ^2^J(H,H) = 15 Hz, ^3^J(H,H) = 5 Hz, H, H2′), 4.84 (dd, ^2^J(H,H) = 15 Hz, ^3^J(H,H) = 5 Hz, H, H2′), 5.72–5.77 (m, 2H, Hb), 5.92–5.97 (m, 2H, Hc), 7.16–7.81 (m, 14H, H4, H5, H6, H7, H4′, H5′, H6′, H7′, H8′, H9′, H10′, H11′), 8.08 (s, 1H, NH-HInd), 9.17 (t, ^3^J(H,H) = 6 Hz, 1H, NH); ^13^C-NMR (125.75 MHz, MeOD) δ: 16.5 (CH_3_-g), 21.0 and 21.2 (2CH_3_-f), 30.7 (Ce), 43.2 (C2′), 80.5 and 80.6 (Cb), 84.2 and 84.3 (Cc), 97.0 (C2), 99.6 (Cd), 102.7 (Ca), 109.6 (C11′), 120.3, 120.5, 120.7, 122.1, 127.0, 127.2, 128.5, 128.7, 132.7, 132.8, 137.0 (C4, C5, C6, C7, C4′, C5′, C6′, C7′, C8′, C9′, C10′), 135.1, 136.9, 139.2, 141.9 (C3a, C7a, C3′, C11a’), 165.5 (C1′), 190.8 (C3), 192.1 (C1); *m*/*z* 631.140, m^th^: 631.149; elemental analysis calcd. for C_34_H_32_N_3_O_3_RuPF_6_·0.5H_2_O: C, 51.98; H, 4.23; N 5.35%; found: C, 51.85; H, 4.24; N, 5.50%.

[(κN-Pyridine)(1,3-dioxo-κO1-1H-inden-2(3H)-ylidene)(benzylamino)methanolato-κO2)](*η*^6^-p-cymene)ruthenium(II) hexafluorophosphate (complex 1f)

The synthesis was performed according to the general procedure for ligand exchange, using complex 1a (60 mg, 110 µmol), silver nitrate (26 mg, 150 µmol), pyridine (11 µL, 130 µmol), and sodium hexafluorophosphate (23 mg, 140 µmol), yielding yellow crystals (72 mg, 89%). m.p.: >174 °C (decomp.), solubility: 0.35 mg/mL = 0.47 mM (MEM, 1% DMSO); ^1^H-NMR (500.10 MHz, MeOD) δ: 1.31 (d, ^3^J(H,H) = 7 Hz, 3H, CH_3_-f), 1.33 (d, ^3^J(H,H) = 7 Hz, 3H, CH_3_-f), 2.11 (s, 3H, CH_3_-g), 2.75–2.81 (m, 1H, He), 4.64 (dd, ^2^J(H,H) = 15 Hz, ^3^J(H,H) = 5 Hz, H, H2′), 4.83 (dd, ^2^J(H,H) = 15 Hz, ^3^J(H,H) = 5 Hz, H, H2′), 5.62–5.69 (m, 2H, Hb), 5.81–5.87 (m, 2H, Hc), 7.33–7.70 (m, 11H, H4, H5, H6, H7, H4′, H5′, H6′, H8′), 7.88–7.92 (m, 1H, H9′), 8.22–8.24 (m, 2H, H7′), 9.13 (t, ^3^J(H,H) = 6 Hz, 1H, NH); ^13^C-NMR (125.75 MHz, MeOD) δ: 17.6 (CH_3_-g), 22.3 and 22.6 (2CH_3_-f), 32.0 (Ce), 44.5 (C2′), 83.0 and 83.2 (Cb), 84.5 and 84.8 (Cc), 98.3 (C2), 99.4 (Cd), 104.7 (Ca), 121.9 (C4, C7), 127.3 (C5, C6), 128.4 and 128.5 (C4′, C6′), 130.0 (C5′), 134.2 (C8′), 136.4 (C7a), 138.4 (C3a), 140.5 (C3′), 140.7 (C9′), 153.4 (C7′), 166.7 (C1′), 191.9 (C3), 193.4 (C1); *m*/*z* 593.138, m^th^: 593.138; elemental analysis calcd. for C_32_H_31_N_2_O_3_RuPF_6_: C, 52.10; H, 4.24; N, 3.80%; found: C, 51.83; H, 4.28; N, 3.78%.

[(κN-Morpholine)(1,3-dioxo-κO1-1H-inden-2(3H)-ylidene)(benzylamino)methanolato-κO2)](η6-p-cymene)ruthenium(II) hexafluorophosphate (complex 1g)

The synthesis was performed according to the general procedure for ligand exchange, using complex 1a (110 mg, 200 µmol), silver nitrate (48 mg, 280 µmol), morpholine (21 µL, 240 µmol), and sodium hexafluorophosphate (42 mg, 260 µmol), yielding a green powder (85 mg, 57%). m.p.: >155 °C (decomp.), solubility: 0.22 mg/mL = 0.29 mM (MEM, 1% DMSO); ^1^H-NMR (500.10 MHz, MeOD) δ: 1.34 (d, 2J(H,H) = 7 Hz, 6H, 2CH_3_-f), 2.13 (s, 3H, CH_3_-g), 2.54–2.60 (m, 1H, H7′), 2.66–2.874 (m, 1H, He), 2.90–2.95 (m, 1H, H7′), 3.10–3.20 (m, 1H, H7′), 3.24–3.31 (m, 1H, H7′), 3.40–3.48 (m, 1H, H8′), 3.51–3.62 (m, 2H, H8′), 3.65–3.70 (m, 1H, H8′), 3.83–3.86 (m, 1H, NH-Morph), 4.67 (d, ^2^J(H,H) = 15 Hz, H, H2′), 4.78 (d, ^2^J(H,H) = 15 Hz, H, H2′), 5.61–5.70 (m, 2H, Hb), 5.78–5.81 (m, 2H, Hc), 7.25–7.67 (m, 9H, H4, H5, H6, H7, H4′, H5′, H6′), 9.27 (t, ^3^J(H,H) = 6 Hz, 1H, NH); ^13^C-NMR (125.75 MHz, MeOD) δ: 15.9 (CH_3_-g), 20.7 and 20.9 (2CH_3_-f), 30.5 (Ce), 42.9 (C2′), 51.8 and 52.5 (C7′), 66.7 and 66.9 (C8′), 79.7 and 80.2 (Cb), 83.8 and 84.0 (Cc), 93.6 (C2), 99.6 (Cd), 103.0 (Ca), 120.5 and 120.6 (C4, C7), 126.3 (C5, C6), 127.0 (C6′), 128.4 (C4′), 132.9 (C5′), 135.5 (C7a), 137.2 (C3a), 138.8 (C3′), 164.0 (C1′), 191.9 (C3), 194.9 (C1); *m*/*z* 601.163, m^th^: 601.164; elemental analysis calcd. for C_31_H_35_N_2_O_4_RuPF_6_·0.5H_2_O: C, 49.34; H, 4.81; N, 3.71%; found: C, 49.51; H, 4.71; N, 3.79%.

### 3.4. Methods

The capacity factors *k* were determined with the use of an RP-UHPLC. The necessary chromatograms were recorded with a Dionex UltiMate 3000 RS UHPLC system (Thermo Fisher Scientific, Bedford, MA, USA) controlled by the Dionex Chromeleon 7.2 software. The following conditions were utilized: mobile phase: MeOH/0.1% formic acid (FA) in water (Milli-Q, 18.2 MΩ·cm, Milli-Q Advantage A10, Darmstadt, Germany); column temperature, 25 °C; flow rate, 0.6 mL/min; UV-VIS detection set at 210, 225, 250, and 320 nm; samples of complexes 1a–g: 1 mM in MeOH; V_inj_ = 5 µL; external dead time marker KI: 0.5 mM in MeOH:water (0.1% FA) (1:1), V_inj_ = 0.8 µL; all samples were filtered through a 0.45 µM regenerated cellulose membrane filter (Minisart RC 4, Sartorius AG, Göttingen, Germany) prior to injection. The capacity factors were then calculated as logk = log[(t_R_ − t_0_)/t_0_], where t_R_ and t_0_ are the retention times of the analyte and the dead time marker, respectively, at the respective MeOH:0.1% FA in water ratio. Every retention time was determined in isocratic mode for the different eluent ratios to obtain capacity factors in the linear range (−0.5 ≤ log*k* ≤ 1.5) [27] in triplicate. A plot of log*k* against the % of the organic eluent yielded logk_w_ (intercept) and S (slope). Φ_0_, the factor most closely resembling log*P*, was then determined as follows: Φ_0_ = −log*k*_w_/S [28].

Stability measurements were performed on a Lambda 35 UV/VIS spectrometer (Perkin Elmer, Boston, MA, USA). Samples of complexes 1a–g: 40 µM in PBS (pH 7.4) and isotonic saline phosphate buffer with 0.9% sodium chloride (pH 8.5), diluted isotonic acetic acid (pH 5.5), and acetate buffer (pH 6.5) with 1% DMSO. UV-VIS spectra were recorded hourly for 24 h at 37 °C. Temperature control was maintained by a PTP-6 Peltier System (Perkin Elmer, Boston, MA, USA). 

Light activation studies were performed on the same UV-VIS spectrometer. Fourty microliters of solutions (MeOH) were irradiated with a conventional desk lamp, and every 2.5–10 min (depending on progress) a wavelength scan (250–700 nm) was recorded.

CH1/PA-1 (ovarian teratocarcinoma) cells were a generous gift from Lloyd R. Kelland (CRC Centre for Cancer Therapeutics, Institute of Cancer Research, Sutton, UK). A549 (non-small cell lung cancer), SW480, and HCT116 (both colon cancer) cells were kindly provided by the Institute of Cancer Research, Department of Medicine I, Medical University of Vienna, Austria. All cell lines were grown as monolayer cultures in Eagle’s MEM supplemented with L-glutamine (4 mM), sodium pyruvate (1 mM) and 1% (*v*/*v*) non-essential amino acid solution (all from Sigma-Aldrich) and 10% (*v*/*v*) heat-inactivated fetal calf serum (FCS, BioWest) in 75-cm^2^ flasks (Starlab) at 37 °C under a humidified atmosphere containing 5% CO_2_ in air.

Antiproliferative activity of the compounds was determined with the colorimetric MTT assay (MTT = 3-(4,5-dimethyl-2-thiazolyl)-2,5-diphenyl-2H-tetrazolium bromide). CH1/PA-1, HCT116, SW480, and A549 cells with cell numbers of 1 × 10^3^, 1.5 × 10^3^, 2 × 10^3^, and 3 × 10^3^, respectively, were seeded in 100 μL per well into 96-well microculture plates (Starlab). After 24 h, test compounds were dissolved in DMSO (Fisher Scientific), serially diluted in complete MEM not to exceed a final DMSO content of 0.5% (*v*/*v*) and added in 100 μL per well. After 96 h, the drug-containing medium was replaced with 100 µL of RPMI 1640/MTT mixture, i. e., 6 parts of RPMI 1640 medium (Sigma-Aldrich; supplemented with 10% heat-inactivated fetal bovine serum and 4 mM l-glutamine), 1 part of MTT solution in phosphate-buffered saline (5 mg/mL; both from Sigma-Aldrich). After incubation for 4 h, the MTT-containing medium was replaced with 150 μL DMSO per well to dissolve the formazan product formed by viable cells. Optical densities at 550 nm (and at the reference wavelength of 690 nm) were measured with a microplate reader (ELx808, Bio-Tek). The IC_50_ values relative to untreated controls were interpolated from concentration–effect curves. At least three independent experiments were performed, each with triplicates per concentration level.

Cellular accumulation of the compounds was studied based on a previously described method [39] with modifications as follows: SW480 cells with a number of 1.8 × 10^5^ per well were seeded into 12-well plates in aliquots of 1 mL complete MEM and incubated at 37 °C for 24 h. Then, cells were exposed for 2 h at 37 °C to 50 μM solutions of the test compounds (containing 0.5% DMSO) in 500 µL of fresh complete MEM upon exchange of the medium. Afterwards, cells were washed three times with PBS, and lyzed with 0.4 mL sub-boiled HNO_3_ per well for 1 h at room temperature. Ruthenium content was quantified by ICP-MS using an Agilent 7500ce quadrupole ICP-MS instrument (Agilent Technologies, Waldbronn, Germany) equipped with a CETAC ASX-520 autosampler (Nebraska, USA) and a MicroMist nebulizer. Ruthenium and indium standards were obtained from CPI International (Amsterdam, The Netherlands). The instrument was equipped with nickel cones and operated at an RF power of 1560 W, with argon as the plasma gas (15 L/min), carrier gas (1.0–1.1 L/min) and make-up gas (0.1–0.2 L/min). The Agilent MassHunter software package (Workstation Software, Version B.01.01, Build 123.11, Patch 4, 2012) was used for data processing. Results are based on at least three independent experiments, each comprising triplicate samples. The instrument was tuned on a daily basis for maximum sensitivity and doubly charged species as well as oxide rates were <5%.

## 4. Conclusions

Starting from the reported ligand L1 and the chlorido complex 1a, six complexes with different *N*-donor ligands (methylimidazole, pyrazole, benzimidazole, indazole, pyridine, and morpholine) were synthesized and characterized via 1D and 2D-NMR spectroscopy, elemental analysis, and in five cases by X-ray diffraction studies. The introduction of these leaving groups increased the solubility in all cases but one. The isolated compounds were found to be highly stable under physiological conditions (pH 7.4–8.5) and might therefore reach the tumor tissue intact. Under the hypoxic and slightly acidic conditions of the tumor tissue, they may be activated by protonation of the *N*-donor and subsequent aquation. Since all compounds are stable at the highest pH occurring in the intestinal tract, they could potentially be administered orally (in a form resistant to gastric acid). Within the tumor tissue, they can be activated by aquation to their corresponding and more reactive aqua complex, which is then able to interact with biological targets. The MTT assay and assessment of the lipophilicity of the obtained complexes showed only little variation.

## Figures and Tables

**Table 1:** Selected bond lengths (Å) and angles (°).

	1a [25]	1b (MeIm)	1c (HPz)	1d (BIm)	1e (HInd)	1f (Pyr)
Ru-O1	2.107(2)	2.086(2) 2.085(2)	2.077(2)	2.073(2)	2.0751(9)	2.0786(15) 2.0847(15) 2.0775(15)
Ru-O2	2.080(2)	2.098(2) 2.104(2)	2.095(2)	2.097(2)	2.0893(9)	2.0948(15) 2.0970(14) 2.0923(14)
Ru-N2 (Cl)	2.419(8)	2.093(2) 2.091(2)	2.099(3)	2.124(4)	2.0979(10)	2.1151(18) 2.1090(18) 2.1094(18)
O1-Ru-O2	90.0(8)	88.76(8) 89.37(8)	89.79(9)	90.16(7)	90.07(3)	89.96(6) 89.71(6) 90.10(6)
C1-O1-Ru-O2	8.92(2)	1.60(5)	4.68(2)	3.14(9)	7.28(6)	7.79(2)
Ru-Cym_centr_	1.639	1.654 1.656	1.660	1.657	1.656	1.657 1.657 1.657
C1-O1	1.267(3)	1.267(3) 1.267(3)	1.270(4)	1.260(3)	1.2637(15)	1.268(3) 1.270(3) 1.263(3)
C3-O3	1.222(3)	1.234(4) 1.228(4)	1.268(4)	1.275(3)	1.2328(17)	1.227(3) 1.228(3) 1.231(3)
Slippage	0.032	0.025 0.028	0.024	0.025	0.017	0.016 0.012 0.008

**Table 2:** Lipophilicity parameters for complexes 1a–g.

	Intercept log*k*_w_	σ	R^2^	Slope S	Φ_0_
1a	5.41	0.0747	0.9997	−0.0855	63.19
1b	5.49	0.0083	0.9996	−0.0888	61.84
1c	5.36	0.0234	0.9996	−0.0841	63.72
1d	5.55	0.0405	0.9983	−0.0843	65.87
1e	5.94	0.0220	0.9999	−0.0863	68.88
1f	5.25	0.0636	0.9994	−0.0845	62.07
1g	5.14	0.0278	0.9999	–0.0860	59.73

**Table 3:** Inhibition of cancer cell growth in four human cancer cell lines; 50% inhibitory concentrations (IC_50_) (µM; means ± standard deviations), obtained by the MTT assay (exposure time: 96 h) and cellular accumulation assessed by inductively coupled plasma mass spectrometry (ICP-MS) quantification of ruthenium (or platinum in the case of cisplatin) in SW480 cells (exposure time: 2 h).

	CH1/PA-1	SW480	HCT116	A549	fg Ru or Pt/Cell
1a	12.5 ± 4.4	12.4 ± 0.4	24.8 ± 6.4	23.4 ± 2.5	8.4 ± 2.8
1b	16.2 ± 2.6	10.9 ± 1.0	24.6 ± 8.5	21.4 ± 1.7	97 ± 28
1c	11.5 ± 2.0	15.2 ± 0.6	22.4 ± 6.0	22.9 ± 3.5	67 ± 27
1d	10.6 ± 1.8	8.8 ± 1.3	18.8 ± 5.8	19.0 ± 0.8	405 ± 128
1e	11.0 ± 3.0	16.8 ± 3.1	25.1 ± 7.1	23.7 ± 6.4	114 ± 35
1f	8.2 ± 2.0	6.3 ± 1.1	20.2 ± 6.4	22.1 ± 3.2	231 ± 93
1g	12.2 ± 2.7	9.2 ± 1.8	23.8 ± 7.6	18.9 ± 1.4	123 ± 32
**Cisplatin ^a^**	0.077 ± 0.006	3.3 ± 0.2		6.2 ± 1.2	12 ± 2

^a^ IC_50_ from ref. [31] and cellular platinum accumulation from ref. [32].

## Supplementary Materials

The following data are available online, Chart S1. ORTEP-representations of 1b–f, Table S1. X-ray data for 1b, Table S2. Data collection and structure refinement of 1b, Table S3. Sample and crystal data of 1c, Table S4. Data collection and structure refinement of 1c, Table S5. Sample and crystal data of 1d, Table S6. Data collection and structure refinement of 1d, Table S7. Sample and crystal data of 1e, Table S8. Data collection and structure refinement of 1e, Table S9. X-ray data for 1f, Table S10. Data collection and structure refinement of 1f, Table S11. Experimental parameters and CCDC-Codes, Table S12. Death time determination for the relevant MeOH percentages in the eluent, Table S13. Determination of logk for 1a–g in triplicate, Figure S1. UV-Vis spectra of 40 µM 1b and 1c at pH 8.5 in 1% *v*/*v* DMSO/H2O (0.9% NaCl, phosphate buffer) at 37 °C, Figure S2. UV-Vis spectra of 40 µM 1b and 1c at pH 7.4 in 1% *v*/*v* DMSO/PBS at 37 °C, Figure S3. UV-Vis spectra of 40 µM 1e at pH 7.4 in 1% *v*/*v* DMSO/PBS at 37 °C, Figure S4. UV-Vis spectra of 40 µM 1f at pH 6.5 in 1% *v*/*v* DMSO/H2O (acetate buffer) at 37 °C, Figure S1. UV-Vis spectra of 40 µM 1f at pH 5.5 in 1% *v*/*v* DMSO/H_2_O (0.9 % NaCl, AcOH) at 37 °C, Figure S14. Graphical comparison of IC_50_-values, Figure S15. Concentration–effect curves in A549 (top) and CH1/PA-1 (bottom) cells, Figure S2. Concentration–effect curves in HCT116 (top) and SW480 (bottom) cells.
