# Supplementary material for: Fine-Tuning the Activation Mode of an 1,3-Indandione-Based Ruthenium(II)-Cymene Half-Sandwich Complex by Variation of Its Leaving Group
Source: Molecules. 2019 Jun 27;24(13):2373. doi: 10.3390/molecules24132373 (PMC6651387; doi:10.3390/molecules24132373)

SUPPORTING INFORMATION

Fine-tuning the activation mode of an 1,3-indandione-based ruthenium(II)-cymene half-sandwich complex by variation of its leaving group

Stephan Mokesch,[1] Daniela Schwarz,[1] Michaela Hejl,[1] Matthias H. M. Klose,[1] Alexander Roller,[1] Michael A. Jakupec,[1,2] Wolfgang Kandioller,[1,2,*] and Bernhard K. Keppler[1,2]

[1] Institute of Inorganic Chemistry, Faculty of Chemistry, University of Vienna, Waehringer Straße 42, A-1090 Vienna, Austria.
[2] Research Cluster "Translational Cancer Therapy Research", University of Vienna, Waehringer Straße 42, A-1090 Vienna, Austria.
* Correspondence: wolfgang.kandioller@univie.ac.at; Tel.: +43-1-4277-52609

Table of Contents

1 Crystallographic Data 2

2 Lipophilicity 9

3 pH-dependent stability 10

4 Biological experiments 13

1 Crystallographic Data

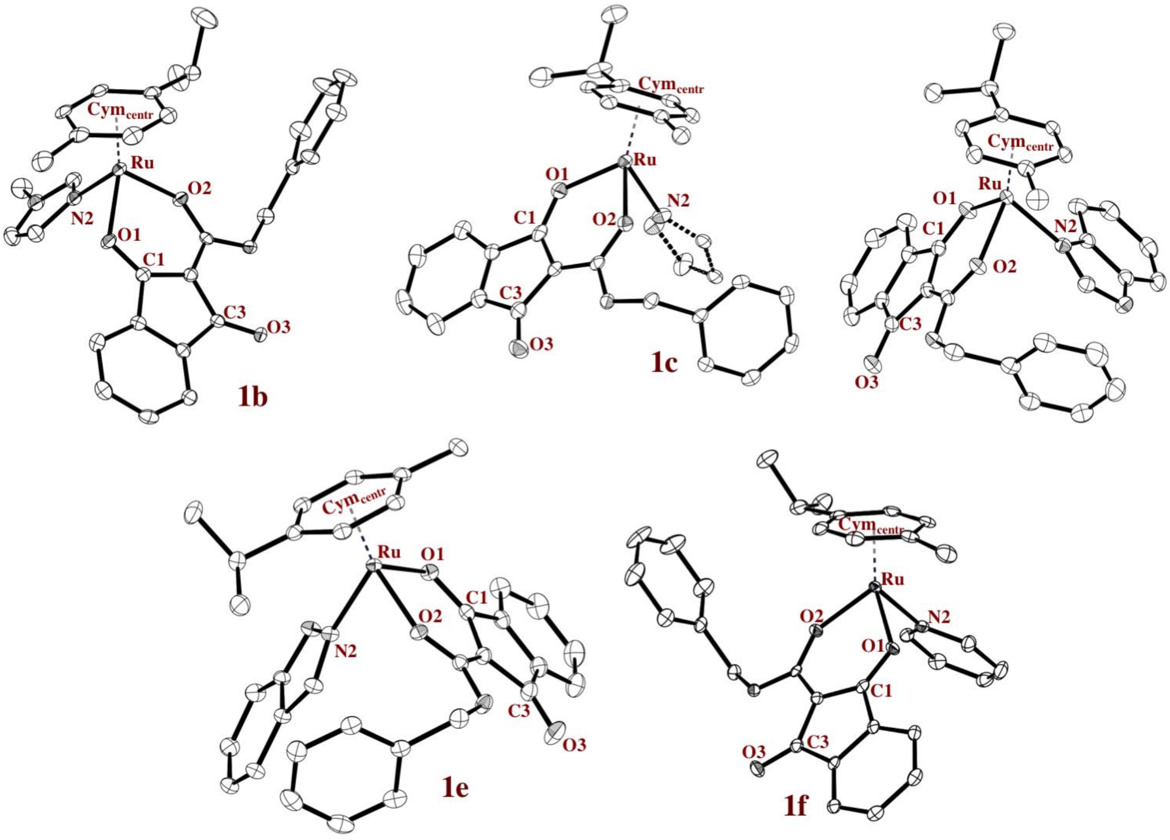

Chart S1. ORTEP-representations of **1b–f**, drawn at the 50% probability level **(**hydrogen atoms and counter ions are omitted for clarity)

Table S1. X-ray data for **1b**

**Chemical formula**	C31H32F6N3O3PRu	**Crystal system**	triclinic	
**Formula weight [g/mol]**	740.63	**Space group**	*P-1*	
**Temperature [K]**	100	**Z**	4	
**Measurement method**	\f and \w scans	**Volume [Å^3]**	3101.83(17)	
**Radiation (Wavelength [Å])**	MoKα (λ = 0.71073)	**Unit cell dimensions [Å] and [°]**	11.6359(4)	70.4432(10)
**Crystal size / [mm^3]**	0.131 × 0.088 × 0.075		15.9779(5)	76.6743(12)
**Crystal habit**	clear yellow block		18.2130(5)	83.2453(11)
**Density (calculated) / [g/cm^3]**	1.586	**Absorption coefficient / [mm^{-1}]**	0.631	
**Abs. correction Tmin**	0.6983	**Abs. correction Tmax**	0.7452	
**Abs. correction type**	multiscan	**F(000) [e$^-$]**	1504	

Table S2. Data collection and structure refinement of **1b**

**Index ranges**	-14 ≤ h ≤ 14, -19 ≤ k ≤ 19, -21 ≤ l ≤ 21	**Theta range for data collection [°]**	2.708 to 50.804	
**Reflections number**	30275	**Data / restraints / parameters**	11390/0/819	
**Refinement method**	Least squares	**Final R indices**	all data	R1 = 0.0483, wR2 = 0.0900
**Function minimized**	Σ w(F_o^2 - F_c^2)^2		I>2σ(I)	R1 = 0.0366, wR2 = 0.0844
**Goodness-of-fit on F^2**	1.033	**Weighting scheme**	w=1/[σ2(Fo2)+(0.0390P)2+5.3443P]	
**Largest diff. peak and hole [e Å^{-3}]**	1.18/-0.69		where P=(F_o^2+2F_c^2)/3	

Table S3. Sample and crystal data of **1c**

**Chemical formula**	C30.5H30F4.5N3O4.5P0.5RuS0.5	**Crystal system**	monoclinic	
**Formula weight [g/mol]**	728.66	**Space group**	*C2/c*	
**Temperature [K]**	100	**Z**	8	
**Measurement method**	\f and \w scans	**Volume [Å^3]**	5944.7(7)	
**Radiation (Wavelength [Å])**	MoKα (λ = 0.71073)	**Unit cell dimensions [Å] and [°]**	18.9427(13)	90
**Crystal size / [mm^3]**	0.4 × 0.25 × 0.02		7.8912(5)	96.668(5)
**Crystal habit**	clear yellow plate		40.040(2)	90
**Density (calculated) / [g/cm^3]**	1.628	**Absorption coefficient / [mm^{-1}]**	0.662	
**Abs. correction Tmin**	0.573	**Abs. correction Tmax**	0.7452	
**Abs. correction type**	multiscan	**F(000) [e$^-$]**	2960	

Table S4. Data collection and structure refinement of **1c**

**Index ranges**	$-22 \le h \le 22$, $-9 \le k \le 9$, $-48 \le l \le 48$	**Theta range for data collection [°]**	5 to 50.698	
**Reflections number**	30444	**Data / restraints / parameters**	5467/6/444	
**Refinement method**	Least squares	**Final R indices**	all data	R1 = 0.0450, wR2 = 0.0891
**Function minimized**	$\Sigma\ w(F_o^2 - F_c^2)^2$		I>2σ(I)	R1 = 0.0426, wR2 = 0.0880
**Goodness-of-fit on F^2**	1.228	**Weighting scheme**	w=1/[σ2(Fo2)+(0.0061P)2+ 37.0240P]	
**Largest diff. peak and hole [e Å^{-3}]**	1.65/-1.20		where $P=(F_o^2+2F_c^2)/3$	

Table S5. Sample and crystal data of **1d**

**Chemical formula**	C34H32F6N3O3PRu	**Crystal system**	monoclinic	
**Formula weight [g/mol]**	776.66	**Space group**	*P21/n*	
**Temperature [K]**	100	**Z**	4	
**Measurement method**	\f and \w scans	**Volume [Å^3]**	3168.46(19)	
**Radiation (Wavelength [Å])**	MoKα (λ = 0.71073)	**Unit cell dimensions [Å] and [°]**	13.9617(5)	90
**Crystal size / [mm^3]**	0.2 × 0.13 × 0.03		16.1368(5)	107.5828(15)
**Crystal habit**	clear yellow plate		14.7527(5)	90
**Density (calculated) / [g/cm^3]**	1.628	**Absorption coefficient / [mm^{-1}]**	0.622	
**Abs. correction Tmin**	0.6975	**Abs. correction Tmax**	0.746	
**Abs. correction type**	multiscan	**F(000) [e$^-$]**	1576	

Table S6. Data collection and structure refinement of **1d**

**Index ranges**	-19 ≤ h ≤ 19, -22 ≤ k ≤ 22, -20 ≤ l ≤ 20	**Theta range for data collection [°]**	4.808 to 60.174	
**Reflections number**	113582	**Data / restraints / parameters**	9288/28/430	
**Refinement method**	Least squares	**Final R indices**	all data	R1 = 0.0588, wR2 = 0.1015
**Function minimized**	Σ w(F_o^2 - F_c^2)^2		I>2σ(I)	R1 = 0.0408, wR2 = 0.0930
**Goodness-of-fit on F^2**	1.02	**Weighting scheme**	w=1/[σ2(Fo2)+(0.0396P)2+ 7.7332P]	
**Largest diff. peak and hole [e Å^{-3}]**	1.42/-1.42		where P=(F_o^2+2F_c^2)/3	

Table S7. Sample and crystal data of **1e**

**Chemical formula**	C35H32F3N3O6RuS	**Crystal system**	monoclinic	
**Formula weight [g/mol]**	780.76	**Space group**	*P21/n*	
**Temperature [K]**	100	**Z**	4	
**Measurement method**	\f and \w scans	**Volume [Å^3]**	3300.7(2)	
**Radiation (Wavelength [Å])**	MoKα (λ = 0.71073)	**Unit cell dimensions [Å] and [°]**	11.2803(5)	90
**Crystal size / [mm^3]**	0.24 × 0.155 × 0.122		16.8126(7)	104.2968(15)
**Crystal habit**	clear yellow block		17.9604(8)	90
**Density (calculated) / [g/cm^3]**	1.571	**Absorption coefficient / [mm^{-1}]**	0.606	
**Abs. correction Tmin**	0.6734	**Abs. correction Tmax**	0.746	
**Abs. correction type**	multiscan	**F(000) [e$^-$]**	1592	

Table S8. Data collection and structure refinement of **1e**

**Index ranges**	-15 ≤ h ≤ 15, -23 ≤ k ≤ 23, -25 ≤ l ≤ 25	**Theta range for data collection [°]**	3.88 to 60.242	
**Reflections number**	126357	**Data / restraints / parameters**	9713/0/445	
**Refinement method**	Least squares	**Final R indices**	all data	R1 = 0.0258, wR2 = 0.0613
**Function minimized**	Σ w(F_o^2 - F_c^2)^2		I>2σ(I)	R1 = 0.0236, wR2 = 0.0597
**Goodness-of-fit on F^2**	1.057	**Weighting scheme**	w=1/[σ2(Fo2)+(0.0274P)2+ 2.5680P]	
**Largest diff. peak and hole [e Å^{-3}]**	0.79/-0.7		where P=(F_o^2+2F_c^2)/3	

Table S9. X-ray data for **1f**

**Chemical formula**	C33H31F3N2O6RuS	**Crystal system**	monoclinic	
**Formula weight [g/mol]**	741.73	**Space group**	*P21/c*	
**Temperature [K]**	100	**Z**	12	
**Measurement method**	\f and \w scans	**Volume [Å^3]**	9532.3(7)	
**Radiation (Wavelength [Å])**	MoKα (λ = 0.71073)	**Unit cell dimensions [Å] and [°]**	19.4630(8)	90
**Crystal size / [mm^3]**	0.395 × 0.22 × 0.052		33.1343(14)	95.0087(15)
**Crystal habit**	clear yellow block		14.8379(7)	90
**Density (calculated) / [g/cm^3]**	1.551	**Absorption coefficient / [mm^{-1}]**	0.624	
**Abs. correction Tmin**	0.5728	**Abs. correction Tmax**	0.746	
**Abs. correction type**	multiscan	**F(000) [e$^-$]**	4536	

Table S10. Data collection and structure refinement of **1f**

**Index ranges**	-21 ≤ h ≤ 23, -39 ≤ k ≤ 39, -17 ≤ l ≤ 17	**Theta range for data collection [°]**	2.1 to 50.7	
**Reflections number**	121329	**Data / restraints / parameters**	17439/0/1252	
**Refinement method**	Least squares	**Final R indices**	all data	R1 = 0.0392, wR2 = 0.0775
**Function minimized**	Σ w(F_o^2 - F_c^2)^2		I>2σ(I)	R1 = 0.0309, wR2 = 0.0737
**Goodness-of-fit on F^2**	1.042	**Weighting scheme**	w=1/[σ2(Fo2)+(0.0289P)2+7.9124P]	
**Largest diff. peak and hole [e Å^{-3}]**	0.88/-0.52		where P=(F_o^2+2F_c^2)/3	

Table S11. Experimental parameters and CCDC-Code.

**Sample**	**Machine**	**Source**	**Temp.**	**Detector Distance**	**Time/ Frame**	**#Frames**	**Frame width**	**CCDC**
	Bruker		[K]	[mm]	[s]		[°]	
**1b**	D8	Mo	100	34	24	686	0.4	1919571
**1c**	X8	Mo	100	50	40	1666	0.5	1919572
**1d**	D8	Mo	100	34	60	2302	0.4	1919573
**1e**	D8	Mo	100	34	30	1654	0.5	1919574
**1f**	D8	Mo	100	35	8	1187	0.4	1919575

2 Lipophilicity

Table S12. Death time determination for the relevant MeOH percentages in the eluent

%	t_0-1	t_0-2	t_0-3	mean
45	0.520	0.520	0.523	0.521
47.5	0.528	0.529	0.528	0.528
50	0.537	0.533	0.533	0.534
52.5	0.533	0.533	0.533	0.533
55	0.530	0.537	0.537	0.535
57.5	0.547	0.547	0.543	0.546
60	0.547	0.553	0.550	0.550

Table S13. Determination of logk for **1a** – **1g** in triplicate

	%	t1	t2	t3	t0	logk1	logk2	logk3
**1a**	52.5	4.837	4.977	4.980	0.533	0.907	0.921	0.921
	50	7.675	7.670	7.650	0.534	1.126	1.126	1.124
	47.5	12.180	12.263	12.160	0.528	1.343	1.347	1.343
**1b**	50	6.583	6.590	6.593	0.534	1.054	1.054	1.055
	47.5	10.325	10.317	10.333	0.528	1.268	1.268	1.269
	45	16.940	16.953	16.907	0.521	1.499	1.499	1.498
**1c**	52.5	5.220	5.283	5.220	0.533	0.944	0.950	0.944
	50	8.082	8.080	8.063	0.534	1.150	1.150	1.149
	47.5	12.807	12.847	12.803	0.528	1.366	1.368	1.366
**1d**	57.5	3.270	3.313	3.277	0.546	0.698	0.705	0.699
	55	5.017	5.080	5.063	0.535	0.923	0.929	0.928
	52.5	7.613	7.600	7.590	0.533	1.123	1.123	1.122
**1e**	60	3.743	3.787	3.733	0.550	0.764	0.770	0.762
	55	9.000	9.120	8.903	0.535	1.200	1.206	1.195
	52.5	14.307	14.275	14.280	0.533	1.412	1.411	1.411
**1f**	52.5	4.047	3.950	3.963	0.533	0.819	0.807	0.809
	50	6.047	6.060	6.060	0.534	1.014	1.015	1.015
	47.5	9.587	9.583	9.590	0.528	1.234	1.234	1.234
**1g**	50	4.197	4.220	4.230	0.534	0.836	0.839	0.840
	47.5	6.427	6.463	6.463	0.528	1.048	1.050	1.050
	45	10.150	10.250	10.150	0.521	1.267	1.271	1.267

3 pH-dependent stability

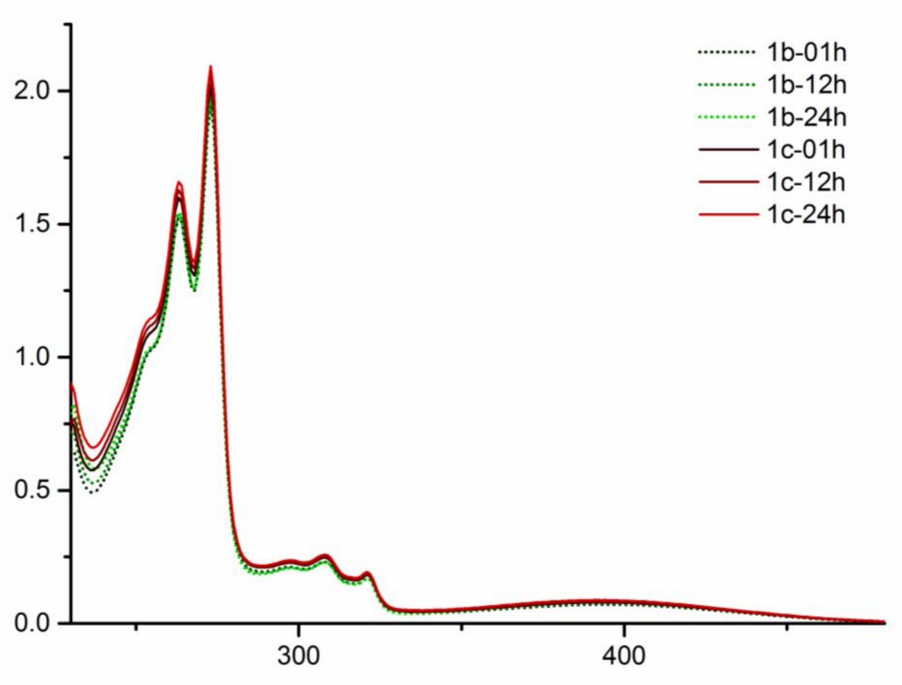

Figure S1. UV-Vis spectra of 40 µM **1b** and **1c** at pH 8.5 in 1% v/v DMSO/H2O (0.9% NaCl, phosphate buffer) at 37 °C

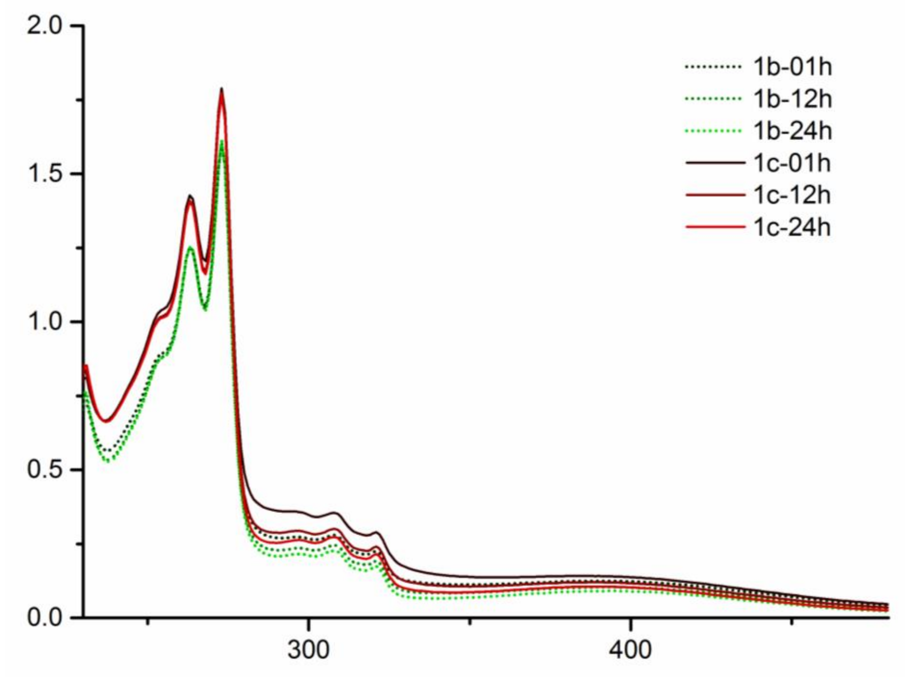

Figure S2. UV-Vis spectra of 40 µM **1b** and **1c** at pH 7.4 in 1% v/v DMSO/PBS at 37 °C

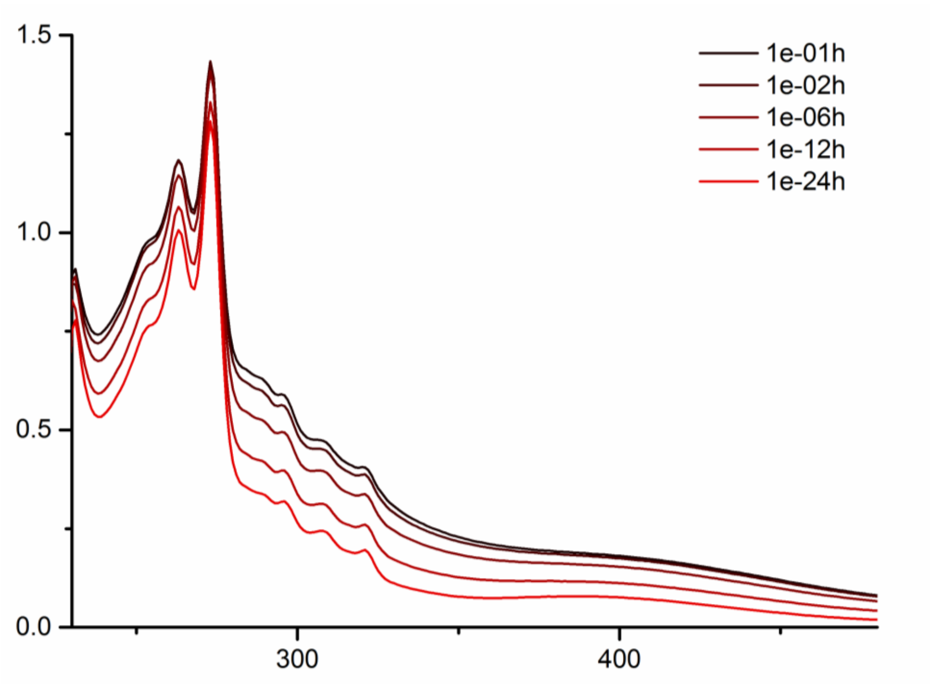

Figure S3. UV-Vis spectra of 40 μM **1e** at pH 7.4 in 1% v/v DMSO/PBS at 37 °C

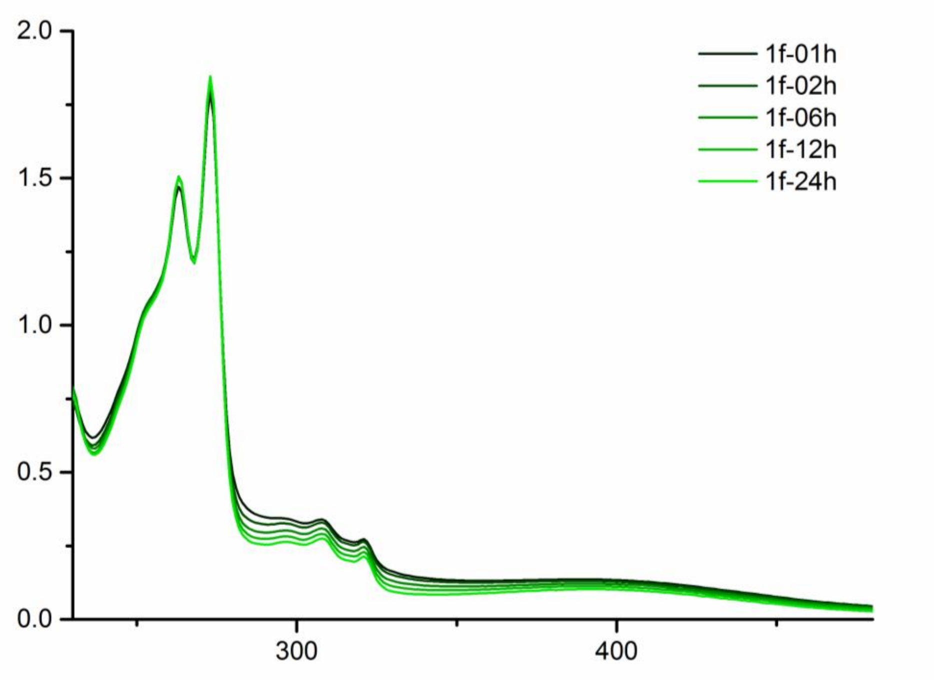

Figure S4. UV-Vis spectra of 40 μM **1f** at pH 6.5 in 1% v/v DMSO/H_2O (acetate buffer) at 37 °C

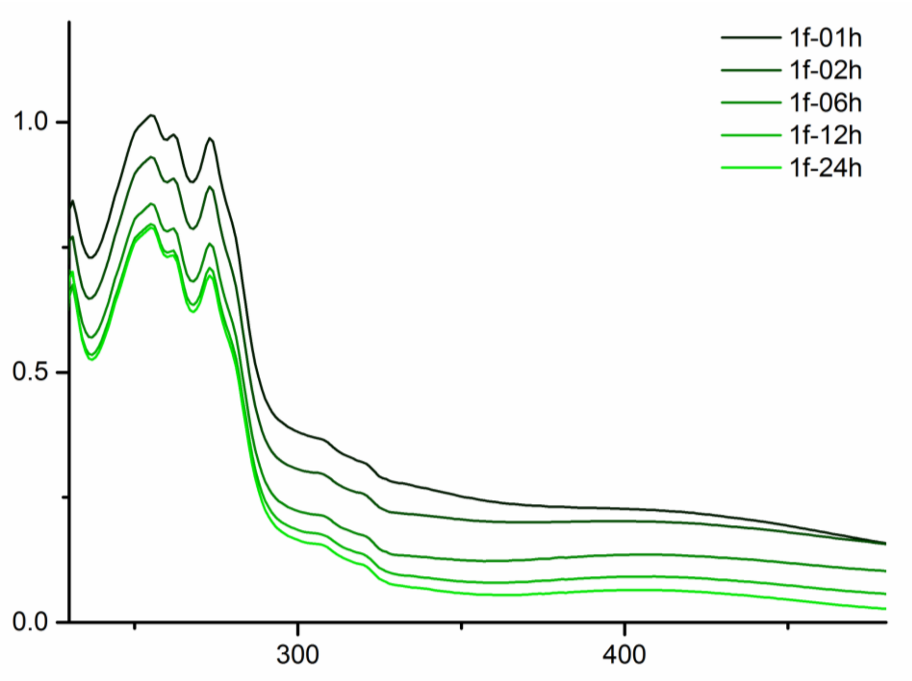

Figure S5. UV-Vis spectra of 40 µM **1f** at pH 5.5 in 1% v/v DMSO/H_2O (0.9 % NaCl, AcOH) at 37 °C

4 Biological experiments

Figure S14. Graphical comparison of IC_{50}-values (complexes are ordered according to their cytotoxicity in the respective cell line)

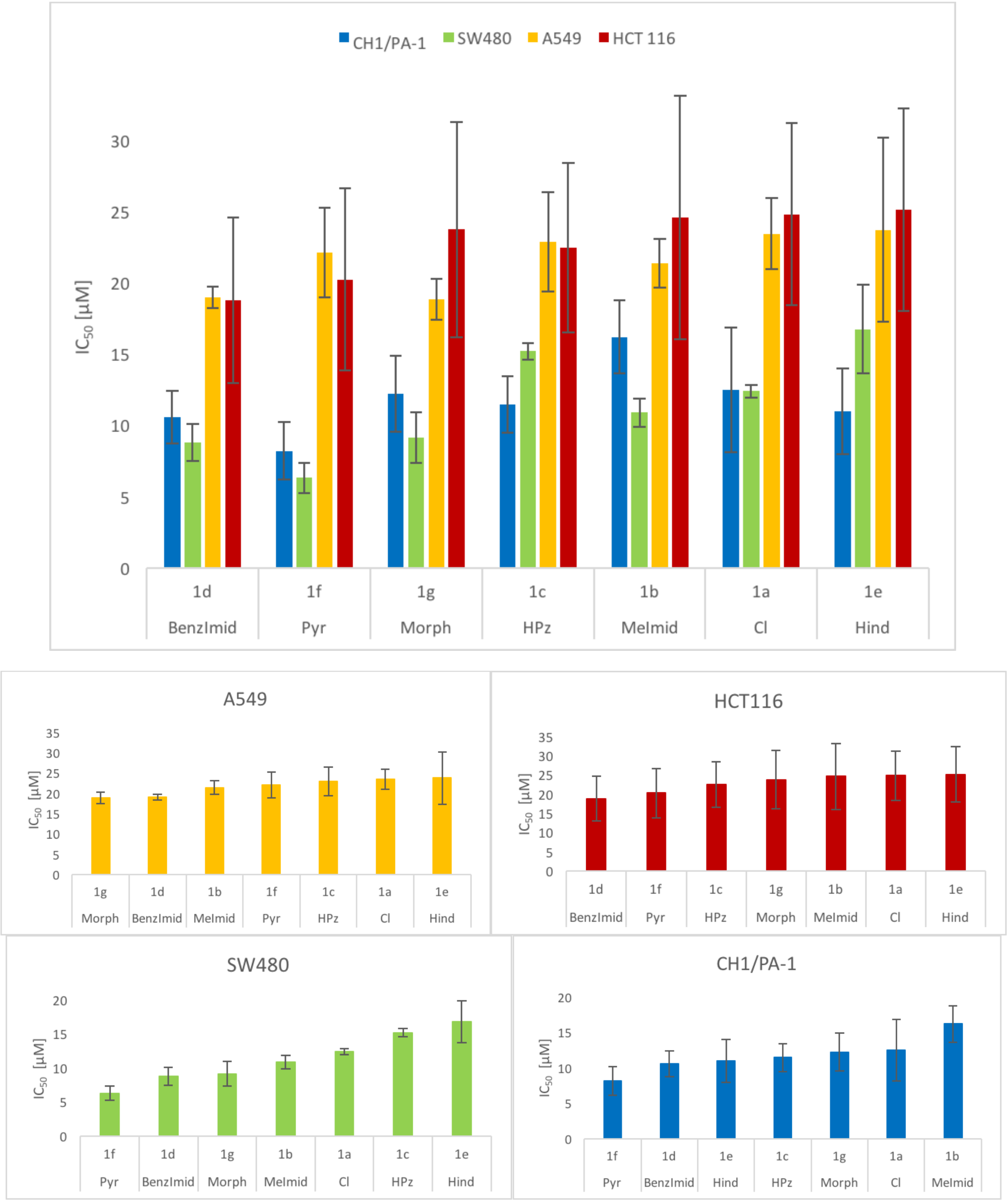

Figure S15. Concentration–effect curves in A549 (top) and CH1/PA-1 (bottom) cells (MTT assay, 96 h exposure)

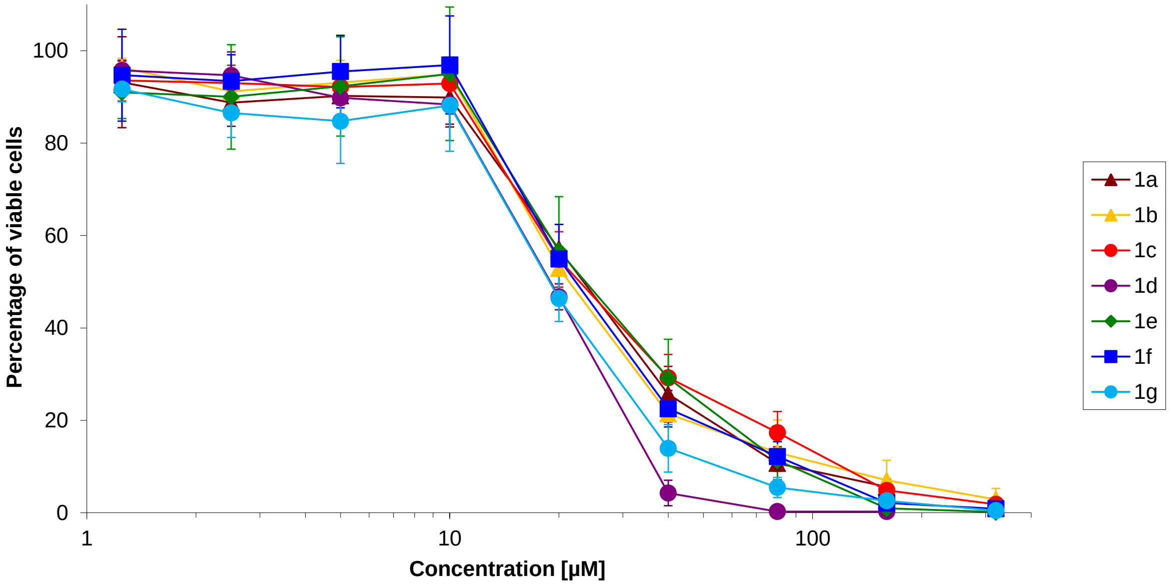

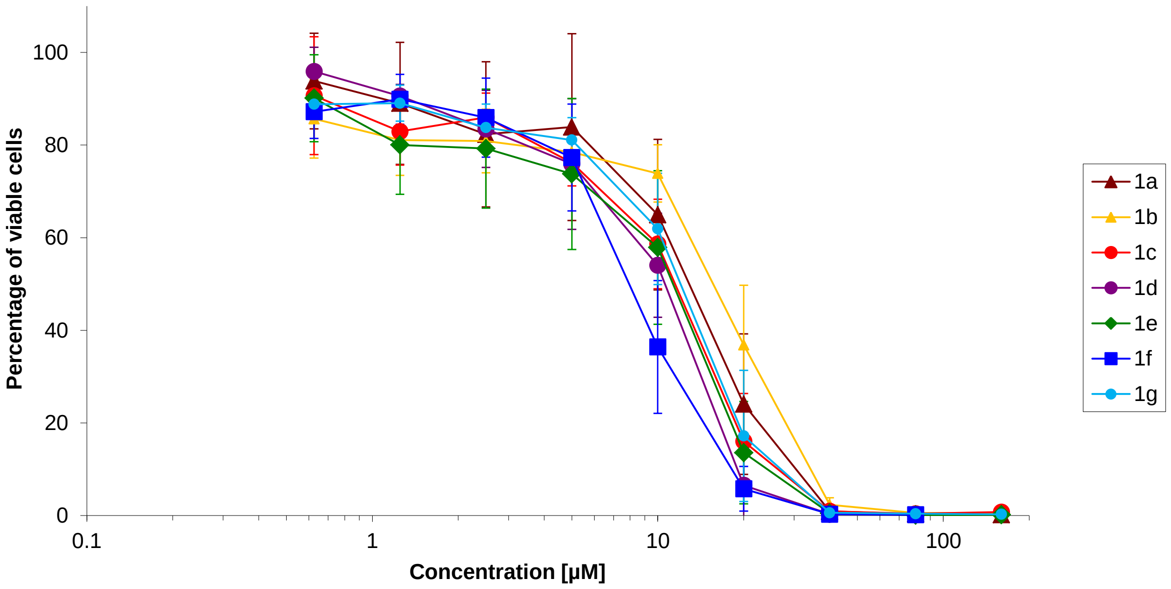

Figure S16. Concentration–effect curves in HCT116 (top) and SW480 (bottom) cells (MTT assay, 96 h exposure)

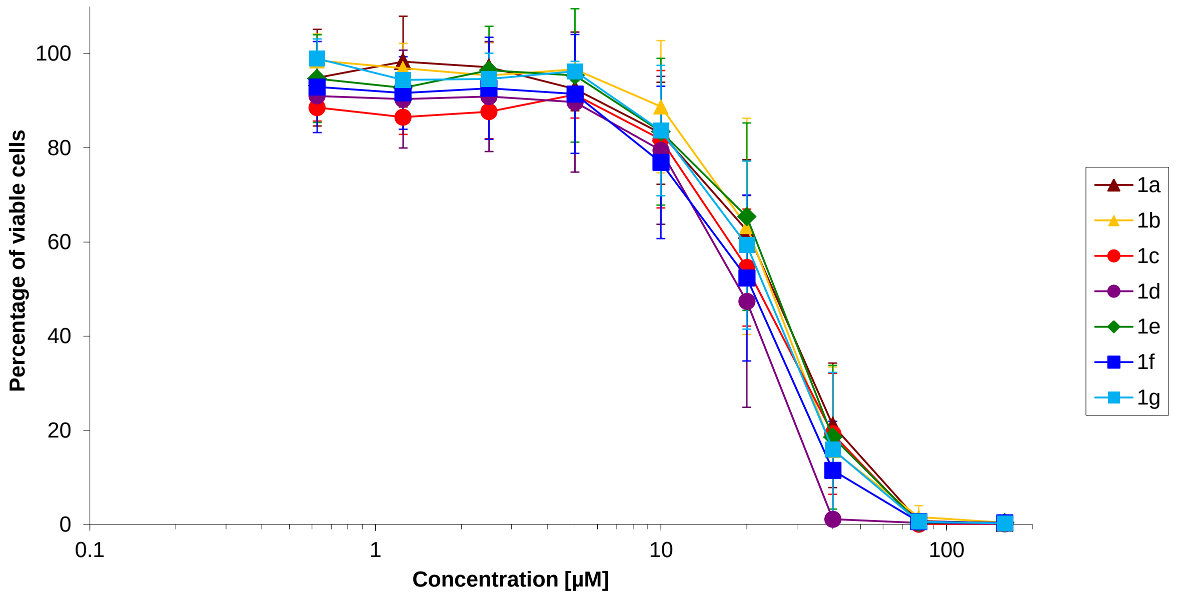

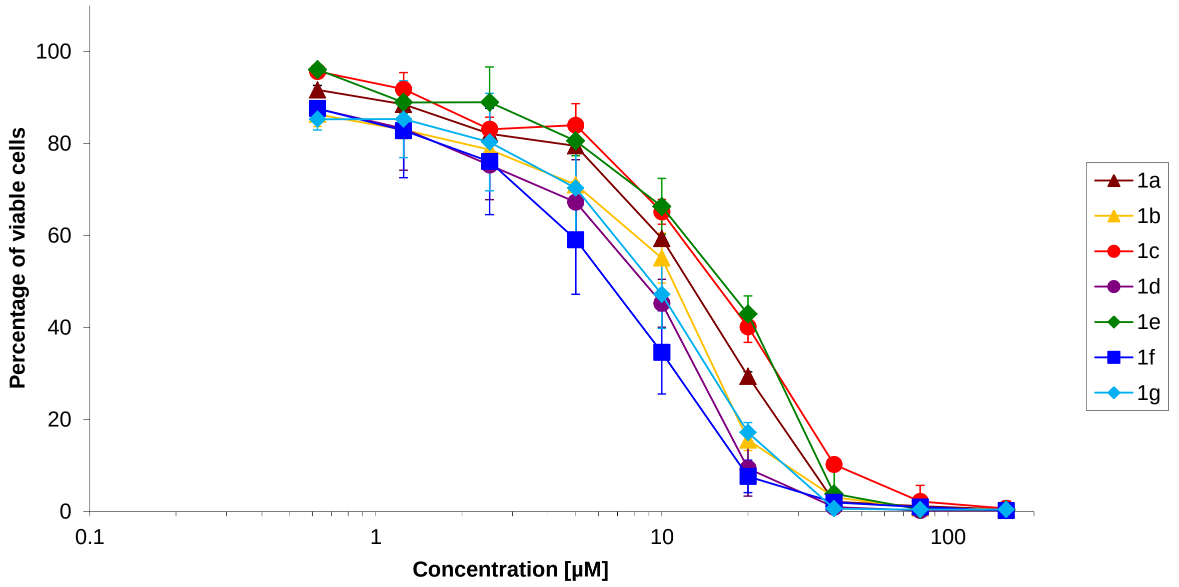

Supplement: Supplementary file 1 [file molecules-24-02373-s001.pdf]
